# Association of Dietary Habits with Eating Disorders among Latvian Youth Aged 18–24

**DOI:** 10.3390/bs14090766

**Published:** 2024-09-02

**Authors:** Inna Gellere, Ilze Beitane

**Affiliations:** Food Institute, Faculty of Agriculture and Food Technology, Latvia University of Life Sciences and Technologies, LV-3004 Jelgava, Latvia; inna.gellere@gmail.com

**Keywords:** SCOFF questionnaire, EDE-Q-6 questionnaire, eating disorder severity, BMI, Canadian Food Intake Screener, Healthy Eating Index

## Abstract

Eating disorders are serious health issues among young people that contribute to increased morbidity rates. The prevalence and severity of eating disorders among Latvian youth aged 18–24 and their relationship with the Healthy Eating Index, dietary habits, and BMI were analyzed in the present study. At the beginning of the study, 190 respondents participated by completing the SCOFF questionnaire, from which the sample group was selected based on the criterion of SCOFF ≥ 2. For the next stage of the study, the responses of 74 participants who completed the EDE-Q-6 questionnaire and the Canadian Food Intake Screener were analyzed. The symptoms of eating disorders were identified in 38.9% of the respondents (SCOFF ≥ 2). The global score of the EDE-Q-6 was 2.00 ± 1.81 where subscales such as body shape concerns (2.93 ± 1.44) and weight concerns (2.68 ± 1.36) were the most important factors that young people paid attention to. The severity of eating disorders was 3.20 for males and 2.62 for females, where a score of 4 and above is classified as an eating disorder. Participants with eating disorder symptoms had a higher BMI, with females having a BMI of 22.5 (normal weight) and 25.5 for males (overweight). The study sample had a poor diet, as the Healthy Eating Index was 8.7 with a maximum score of 24. Weak negative correlations were found between eating disorder severity and eating habits. The issue of eating disorders is topical among young people in Latvia, which requires solutions such as the inclusion of educational programs on healthy nutrition and eating disorders, and psychological support for young people.

## 1. Introduction

Eating disorders are a serious health issue that have a significant impact on morbidity and mortality rates, increasing them several fold [1]. Although statistically, the prevalence of eating disorders (*Anorexia nervosa* and *Bulimia nervosa*) in Europe is below 1% of the population—for example, 0.2% in Latvia, Lithuania, Estonia, and Poland, 0.5% in Germany and France, and 0.6% in Italy and Spain [2]—it is important to detect eating disorders early to minimize the negative effects and progression to more severe consequences [3,4]. The simplest screening tool to identify possible symptoms of eating disorders is the SCOFF questionnaire, which is widely used [5].

Europe ranks third in the prevalence of eating disorders, behind the American continent and Asia, with a global increase of 4.3% from 2000 to 2018 [6]. The European Parliament estimates that 20 million Europeans have an eating disorder [7]. The prevalence of eating disorders is influenced by several factors such as gender, eating problems during childhood, and concerns about weight [8]. In a study of Chinese university students aged 18–24, gender was one of the most significant risk factors for developing eating disorders [9]. The prevalence of eating disorders in women is significantly higher compared to men, with the gender ratio between females and males varying from 3:1 to 10:1 [10]. This is confirmed by the body appreciation results in which 17-year-old boys rate their bodies more positively than girls of that age [11]. Family relationships (children and parents) also play an essential role, as adolescence is the age with the highest risk of developing eating disorders [12]. It is important to encourage adolescents’ involvement in family meals to create a positive eating experience [13] because unhealthy food consumption and meal skipping are common among adolescents [14]. Studies show that the place of residence also has a significant impact on body shape as urban women are slimmer and less satisfied with their body shape [15], which in turn increases the risk of developing an eating disorder.

The COVID-19 pandemic also played an integral role in the rise of eating disorders. Studies have shown that the increase in eating disorders among adolescents in the USA was recorded to be 15.3% [16], while in Germany, it was 40% among children and adolescents [17]. In their study, Gilsbach et al. stated that the COVID-19 pandemic has contributed to the severity of *Anorexia nervosa* symptoms among children and adolescents in Europe [18].

Studies have reported that food choices not only affect an individual’s weight/health but can also reduce/increase the risk of developing an eating disorder. Dietary restraint is considered a precursor to binge eating [19]. While the cohort study carried out in Spain concluded that Mediterranean nutritional patterns can reduce the risk of developing an eating disorder [20], the study by Ayton and Ibrahim [21] indicated that patients with eating disorders choose more ultra-processed foods. This demonstrates the essential contribution of healthy dietary habits developed in childhood to physical and mental health in adulthood.

Considering that the highest risk for the prevalence of eating disorders among young people is 21 years of age on average [22], the current study sample was made up of young people aged 18–24. The study aims to analyze the prevalence and severity of eating disorders among Latvian youth aged 18–24 and their relationship with the Healthy Eating Index, dietary habits, and BMI.

Previous studies in Latvia have not analyzed the eating habits of young people with symptoms of eating disorders, nor have they evaluated the relationships between the eating habits and the severity of their eating disorders. This research will make it possible to update this question because the problem of eating disorders is not paid enough attention in Latvia.

## 2. Materials and Methods

### 2.1. Study Design

On 20 February 2024, protocol extract no. 18–29/1 was approved by the Research Ethics Committee of the Faculty of Biology and the Faculty of Geography and Earth Sciences of the University of Latvia to conduct this study.

**Figure 1:**
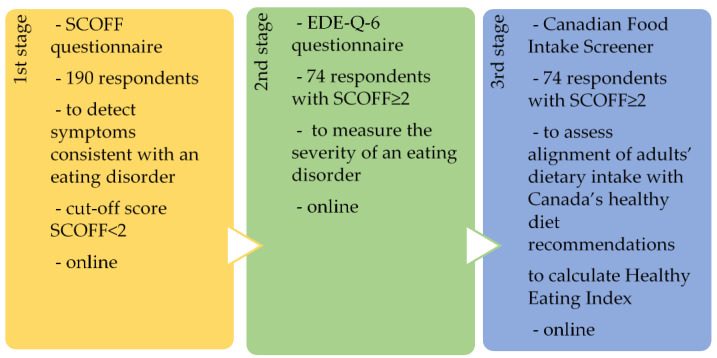
Study design.

The research design was a quantitative study that was carried out in Latvia and the data collection method used three types of surveys: SCOFF (Sick, Control, One, Fat, Food), Eating Disorder Examination Questionnaire 6 (EDE-Q-6), and the Canadian Food Intake Screener (Figure 1).

The questionnaires were distributed online and sent to student self-governments at Rīga Stradiņš University and Latvia University of Life Sciences and Technologies with a request to distribute them among students. Participation in the surveys was voluntary. The questionnaires were translated into Latvian, as the study was aimed at young people residing in Latvia. The criteria for including study participants were age 18 to 24 years, being a resident of Latvia, filling out all three questionnaires, and agreeing to participate in the study. One study participant was excluded due to an incomplete questionnaire.

A total of 190 respondents participated in all three stages of the study, but only those respondents who had provided at least two positive answers out of five in the SCOFF questionnaire were selected for further analyses in stages 2 and 3 of the study because they were in line with the aim of the study. There were 74 respondents. In the second stage, the severity of the respondents’ eating disorders was analyzed with the help of the EDE-Q-6 survey. In the third stage of the study, the respondents’ eating habits were analyzed using the Canadian Food Intake Screener to further evaluate the relationship between eating habits and eating disorder severity.

Participants with positive responses to the SCOFF questionnaire below two did not have symptoms of an eating disorder. Therefore, a decision was made not to further evaluate the questionnaires of these participants regarding the severity of eating disorders and their correlation with eating habits.

### 2.2. Participants

In total, 190 young people aged 18 to 24 participated in the study. The distribution of participants by gender was 137 females and 53 males with mean age 20.51 ± 2.18. Each participant familiarized themselves with the informed consent data before filling out the questionnaires. The questionnaires were filled out electronically on the website https://visidati.lv (accessed on 26 July 2024). Data collection was conducted from the end of February to the end of March 2024.

### 2.3. SCOFF Questionnaire

The SCOFF questionnaire is a screening tool, which helps detect eating disorders [23]. In the first stage of the study, all respondents answered the five questions of the SCOFF questionnaire. Respondents who gave a positive answer (yes) to two or more questions were selected for further comprehensive evaluation. These respondents were identified as having symptoms consistent with an eating disorder. Out of 190 sample respondents, 74 respondents were selected to meet the criteria of the study. Out of the 74 respondents selected, 14 (18.9%) were males. The mean age was 20.68 ± 2.16. The selection was made in the SPSS program by choosing those respondents who answered “yes” to at least 2 questions.

### 2.4. EDE-Q-6 Questionnaire

The Eating Disorder Examination Questionnaire (EDE-Q-6), which focuses on the last 28 days, allows one to measure the severity of an eating disorder [24]. All 28 questions of the questionnaire, except the open-ended questions from 13 to 18, were divided into four subscales: restraint, eating concern, weight concern, and body shape concern. Subscale scores reflect the severity of the aspects of an eating disorder psychopathology. Subscale scores were reported as means and standard deviations. The global score was obtained from the ratings of the four subscales. The answers to the questions were coded according to the Likert scale (from 0 to 6) in the SPSS program. A score of 4 and above is considered a clinically significant range and is classified as an eating disorder.

At the end of the EDE-Q-6 questionnaire, participants indicated their current weight and height. The obtained data were used to calculate Body Mass Index (BMI), according to the following formula: body mass in kilograms divided by height in meters squared. A BMI < 18.5 is classified as underweight, a BMI of 18.5 to 24.9 is considered optimal weight, a BMI of 25.0 to 29.9 is overweight, and a BMI > 30.0 is classified as obesity [25].

### 2.5. Canadian Food Intake Screener

The Canadian Food Intake Screener assesses the alignment of adults’ dietary intake with Canada’s healthy diet recommendations [26]. The survey consists of 16 questions and assesses the frequency of healthy and restricted food consumption in the past month. The respondents had to choose one of ten answer options, ranging from never to six or more times a day. Answers to questions about healthy foods were rated higher when more frequent consumption was indicated, while answers about restricted food were rated higher when less frequent consumption was indicated. The scoring system for the Canadian Food Intake Screener was used according to the Hutchinson et al. [26] study. In order to interpret the results, all questions were divided into categories: (1) vegetables and fruit; (2) whole grain foods; (3) grain foods ratio (whole grain foods/total grain foods); (4) protein foods; (5) plant-based protein foods; (6) unsaturated oils; (7) foods and beverages high in sugars; (8) foods high in sodium/saturated fat. The obtained data were coded in the SPSS program on a scale from 0 to 9, where 0 is the minimum number of points and 9 is the maximum. According to the evaluation method instructions, the number of points for each category was calculated for each respondent as well as the global score which was equated to the Healthy Eating Index.

### 2.6. Statistical Analysis

A descriptive statistical analysis of the study data was carried out with the help of SPSS Statistics 29 and Microsoft Excel v16 programs. Mean values and standard deviation were calculated for the following parameters: the average severity of the eating disorder and the average severity of the eating disorders’ subscales (restraint, eating concern, weight concern, and body shape concern). Mean values were calculated for BMI for the following groups of respondents: all respondents; respondents with eating disorder symptoms; males; males with eating disorder symptoms; females; females with eating disorder symptoms.

The chi-square test was used to analyze the EDE-Q-6 questionnaire data to determine the predominant responses, which made it possible to reveal the psychopathological severity of eating disorders. Hypotheses were formulated for data analysis: H_0_ when the number of respondents’ answers did not differ significantly; H_1_ when the number of respondents’ answers differed significantly. Using statistical analysis χ^2^ the criterion was calculated for each question. Chi-square test values were calculated for questions 1 to 21. Questions 22 to 28 were not included in the analysis because the chi-square test cannot be used if there are too many 0 values.

Correlation coefficient values were calculated between the Healthy Eating Index and the severity of an eating disorder; between the categories of the Canadian Food Intake Screener and the severity of an eating disorder to determine which food group could be related to the severity of an eating disorder; and between BMI and the severity of an eating disorder.

## 3. Results

### 3.1. Results of the SCOFF Questionnaire

**Figure 2:**
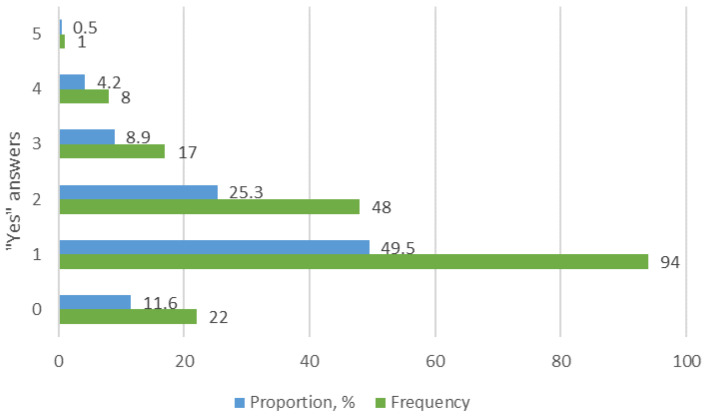
Results of the SCOFF questionnaire.

The analysis of the SCOFF questionnaire selected the respondents who answered “yes” to at least two of the five questions to identify young people with symptoms of an eating disorder (Figure 2). The participants were classified into two groups based on the cut-off score of SCOFF < 2. One group consisted of participants who gave no or one positive answer. This group was excluded from further study. The second group consisted of participants who provided two or more positive answers and was included in the further study.

The results showed that only 11.6% of the 190 respondents answered negatively to all questions, while 49.5% the of respondents answered “yes” to only one question. Therefore, 61.1% of the respondents did not meet the study criteria (cut-off score of SCOFF < 2) and these respondents were excluded from the further sample. The next stage of the study included 74 (38.9%) respondents who answered “yes” to at least two questions because they were observed to exhibit behaviors characteristic of eating disorders.

### 3.2. Statistical Analysis EDE-Q-6 Questionnaire

Descriptive statistics were used to calculate the average severity of eating disorders by subscales for sample respondents (Table 1).

The highest degree of severity was found in the subscale “shape concern”, which indicated that the majority of respondents had a strong concern about their physical appearance, which is one of the predisposing factors for eating disorders. Similar findings were reported in an Australian study where 28% of the general population indicated having increased their food restrictions to change body weight or shape since the COVID-19 pandemic [27].

The average severity of eating disorders was 3.20 for males and 2.62 for females. As a result of the chi-square test, it was found that most of the questions in the EDE-Q-6 questionnaire had dominant responses (Table 2).

The results revealed that the majority of respondents with eating disorder symptoms had concerns about shape or weight, strong concerns about losing control over eating, and a strong desire to diet and lose weight. These concerns about eating and shape or weight, in general, interfered with focusing on daily activities and negatively affected the respondents’ quality of life.

**Figure 3:**
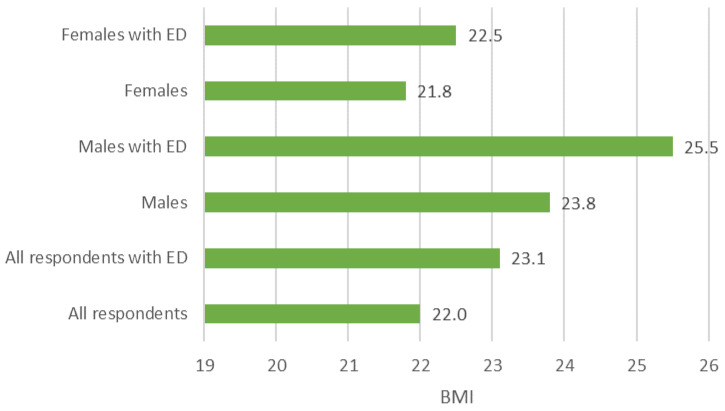
BMI of respondents with and without eating disorder symptoms (ED) by sex.

By evaluating the BMI of the respondents, it can be concluded that the respondents with eating disorder symptoms have a higher BMI (Figure 3). However, the severity of eating disorders did not depend on the BMI (*p* = 0.271). Males with eating disorder symptoms showed an average BMI above 25.0, which is classified as overweight.

### 3.3. Correlation between Adults’ Dietary Intake and Eating Disorder Severity

According to the Canadian Food Intake Screener assessment system, the average Healthy Eating Index of respondents was 8.7 where the maximum score is 24. The results obtained therefore indicated comparatively poor diets among the respondents. When evaluating the correlation between the Healthy Eating Index and eating disorder severity, a weak negative correlation was found. Similar findings were shown in the correlations between eating disorder severity and eating habits (Table 3).

The dietary habits such as consumption of whole grain foods, protein foods, plant-based protein foods, unsaturated oils, foods and beverages high in sugar, and foods high in sodium/saturated fat showed moderate negative correlations with eating disorder severity.

## 4. Discussion

In the initial stage of the study, identifying the symptoms of eating disorders in young people aged 18–24 years, the obtained result of the SCOFF questionnaire was two times higher (38.9%) compared to a study with Irish youth aged 11–19, where only 16.2% (SCOFF ≥ 2) of the respondents who showed the symptoms of eating disorders were selected [28]. In a study of Italian adolescents aged 14–19 years, 31.0% of the participants had a SCOFF score of 3 or more [29]. The essential differences between these studies could be related to the impact of COVID-19, which traumatized people’s mental health, including intensifying eating disorders [30]. A study conducted in Australia reported that 34.6% of the general population showed increased binge eating behavior during the COVID-19 pandemic [27].

In a study with Spanish adolescents, disordered eating was found in 30.1% of adolescents [13], which could be one of the prerequisites for the risk of an eating disorder. In a study with Italian adolescents, disordered eating was also associated with a higher BMI [29], which was also confirmed in the current research and which suggests again a link between overweight and eating disorders.

The global score of the EDE-Q-6 in the present study (2.00 ± 1.81) was higher than the global score reported by Welch et al. [31] for the Australian general population of females (1.56 ± 1.27), and by Baceviciene et al. [32] for Lithuanian students (1.5 ± 1.2). This could be explained by the fact that in the present study, the respondents were selected for further research after having completed the SCOFF questionnaire.

Shape and weight concerns were the most important factors that young people paid attention to. Similar findings were reported in a study of an Australian general population of females, where body shape concern had the highest mean score of 2.40 [31]. In a study of Lithuanian students, the highest score among the subscales was body shape concern with a mean score of 2.0 ± 1.5 [32]. A study of Chinese university students reported a significant correlation between eating disorders and body shape [9]. In a study of adolescents, the most important indicator of eating behavior was weight loss [13]. A Spanish study of young people found that girls with a high risk of eating disorders did more physical activity to lose weight compared to peers with a low risk of developing an eating disorder [33].

The study sample faced daily challenges such as fear of losing control over eating, fear of gaining weight, thoughts about food and being fat, and the desire to lose weight. This in turn affected the eating habits of the sample group, resulting in a low Healthy Eating Index. This indicated that the desire to lose weight and the misinterpretation of what constitutes a healthy diet can lead to eating disorders [34].

The study results confirmed that the healthier one’s eating habits are, the lower the severity of their eating disorder. However, the study sample was characterized by a poor diet as evidenced by a low Healthy Eating Index—8.7. Atypical eating behaviors have been observed in patients with eating disorders, such as skipping meals, binge eating episodes, night eating, nibbling [35], strict eating routines, and rigid, ritualized behavior patterns [36]. Breakfast skipping and evening snacking are eating patterns observed in binge eating patients [37]. Roustaee et al. [38] reported on three dietary patterns observed in female adolescents with eating disorders: high carbohydrate and high fat; high protein and high fat; and high fiber and low fat patterns. High fiber and low fat dietary patterns had higher probabilities of eating disorders. An interesting finding in the present study was a correlation between the severity of an eating disorder and sugar consumption: the higher the consumption of high-sugar products, the lower the severity of the eating disorder. This correlation could be explained by the atypical eating habits in patients with eating disorders, who choose ultra-processed foods such as reduced sugar or zero sugar products/beverages [21]. Johansson et al. [39] reported that patients with eating disorders had a high intake of caffeinated and light soft drinks and a low intake of sweets. When analyzing patients with *Anorexia nervosa*, it was observed that the higher the severity of the eating disorder, the greater the desire for low-calorie products [40]. This also explains the conflicting correlation between the lower consumption of foods high in sodium and saturated fats and higher eating disorder severity. Participants with eating disorder symptoms chose products low in fat and salt.

Some limitations of the study should be noted. First, the questionnaires were distributed and the data were collected online. On the one hand, this allowed us to address a larger audience, but on the other hand, it increased the risk of non-attending participants in the survey. Second, the results were based on the respondents’ self-assessments; therefore, it was not possible to verify the truthfulness and accuracy of the respondents’ answers. Randomized telephone interviews or face-to-face meetings could be a solution to enhance the accuracy of the data collected, but this would, in turn, reduce the sample size. Third, the sample size of the study was relatively small, and repeated studies with a large number of respondents are necessary. To increase the sample group of young people with eating disorder symptoms, it would be necessary to cooperate with practicing nutritionists. Fourth, the study group had a small number of males; therefore, the results of the study cannot be extended to the male population. Targeted sampling is required, using personal appeals to each person to participate in the study; in this case, online surveys cannot provide this.

## 5. Conclusions

The data from the present study show that among 190 young people, there were 74 individuals with symptoms of an eating disorder who had a poor diet and strong concern about their shape, which affected their quality of life. The average severity of eating disorders was 3.20 for males and 2.62 for females, where a score of 4 and above is classified as an eating disorder. A statistically significant part of participants felt fat, were afraid of losing control over eating and gaining weight, desired to lose weight, and were exposed to these feelings every day. Their Healthy Eating Index was 8.7, with a maximum score of 24, and their dietary habits showed moderate negative correlations with eating disorder severity. Males with eating disorder symptoms showed an average BMI above 25.0, which is considered overweight.

Further research is necessary and, simultaneously, it is necessary to talk about the symptoms of eating disorders, explain the principles of a healthy diet to Latvian university students and, if necessary, provide psychological support.

## Figures and Tables

**Table 1:** The severity of eating disorders by subscales.

Subscales	Mean Score ± Standard Deviation
Restraint	2.02 ± 1.21
Eating concern	2.18 ± 1.75
Weight concern	2.68 ± 1.36
Shape concern	2.93 ± 1.44
Global score	2.00 ± 1.81

**Table 2:** Dominant responses to EDE-Q-6 questionnaire.

Questions	H_1_ *	Dominant Response
Have you been deliberately trying to limit the amount of food you eat to influence your shape or weight?	*p* = 0.001	1–5 days
Have you gone for long periods of time without eating anything at all in order to influence your shape or weight?	*p* = 0.001	No days
Have you tried to exclude from your diet any foods that you like in order to influence your shape or weight?	*p* = 0.001	1–5 days
Have you tried to follow definite rules regarding your eating in order to influence your shape or weight?	*p* = 0.001	1–5 days
Have you had a definite desire to have an empty stomach with the aim of influencing your shape or weight?	*p* = 0.001	No days
Have you had a definite desire to have a totally flat stomach?	*p* = 0.001	No days
Has thinking about food, eating or calories made it very difficult to concentrate on things you are interested in?	*p* = 0.001	Every day
Has thinking about shape or weight made it very difficult to concentrate on things you are interested in?	*p* = 0.001	No days
Have you had a definite fear of losing control over eating?	*p* = 0.001	Every day
Have you had a definite fear that you might gain weight?	*p* = 0.001	Every day
Have you felt fat?	*p* = 0.001	Every day
Have you had a strong desire to lose weight?	*p* = 0.001	Every day
Over the past 28 days, on how many days have you eaten in secret?	*p* = 0.001	No days
On what proportion of the times that you have eaten have you felt guilty because of its effect on your shape or weight?	*p* = 0.001	No days
Over the past 28 days, how concerned have you been about other people seeing you eat?	*p* = 0.001	No days

* H_1_: the number of respondents’ answers differs significantly.

**Table 3:** Correlation between eating disorder severity and eating habits.

Indicators	Correlation Coefficient	Coefficient of Determination, R^2^
Healthy Eating Index and ED severity	r = −0.53	0.2837
ED severity and consumption of vegetables and fruits	r = −0.48	0.2257
ED severity and consumption of whole grain foods	r = −0.58	0.3393
ED severity and consumption of protein foods	r = −0.55	0.3029
ED severity and consumption of plant-based protein foods	r = −0.53	0.2827
ED severity and consumption of unsaturated oils	r = −0.55	0.2984
ED severity and consumption of foods and beverages high in sugars	r = −0.61	0.1524
ED severity and consumption of foods high in sodium/saturated fat	r = −0.50	0.2474

ED—eating disorder.

## Data Availability

The data obtained in this study are available on request from the corresponding author.
